# OA03 “I can't stand this pain anymore. Please chop my leg off.” Is amputation a therapeutic option for Complex Regional Pain Syndrome in children and young people?

**DOI:** 10.1093/rap/rkac066.003

**Published:** 2022-09-28

**Authors:** Valerie Rogers, Hannah Connell, Karen Davies, Jeremy Gauntlett-Gilbert

**Affiliations:** Department of Paediatric Rheumatology, University of Bristol and Weston NHS Trust, Bristol, United Kingdom; Bath Centre for Pain Services, Bath, United Kingdom; Bath Centre for Pain Services, Bath, United Kingdom; Department of paediatric Rheumatology, The Royal Wolverhampton NHS Trust, Wolverhampton, United Kingdom; Bath Centre for Pain Services, Bath, United Kingdom

## Abstract

**Introduction/Background:**

Complex Regional Pain Syndrome (CRPS) is a chronic intensified localised pain condition. Symptoms may include limb pain, allodynia; hyperalgesia; swelling and/or changes in skin colour of the affected limb; dry, mottled skin; hyperhidrosis and trophic changes of the nails and hair. There is no known cure.  Management involves analgesic medication and intensive multidisciplinary pain rehabilitation.  Some cases however seem intractable, with little or no response to therapy.  Some patients feel amputation of the affected limb is the only possible option.  We present four cases of children and young adults requesting amputation for CRPS.

**Description/Method:**

Case 1: 13 years, CRPS left leg, with associated gross oedema, ulceration and high risk of sepsis.

Case 2: 16 years, CRPS both feet and ankles, ulceration, fixed dystonic posturing bilaterally, Autistic Spectrum Disorder

Case 3: 17 years, mild cerebral palsy affecting left side, passionate about sport, development of CRPS following surgery for tendon release right hip flexors, fixed flexion contractures at right hip and knee.

Case 4:17 years, high functioning athlete, CRPS initially foot and ankle, contractures ankle, knee and hip; progression to involve other leg.

In each of these cases, prolonged intensive therapeutic interventions have not been successful in restoring function or reducing pain. All have requested amputation of the affected limb(s).

**Discussion/Results:**

The tabloid press has reported the transforming success of amputation for some cases of CRPS, describing individuals who have had CRPS which has not responded to treatment finally having the affected limb amputated, and subsequently achieving great things - including winning medals in the Para-Olympics.  Unsurprisingly such success stories provide hope for some enduring CRPS and becomes part of their ‘evidence’ for requesting amputation.

The scientific literature however is sparce, and virtually non-existent in children and young people.  The risks of ongoing pain in the stump; recurrence of CRPS in the amputated limb - or elsewhere; phantom limb pain and inability to tolerate a prosthesis are well documented in the adult literature that is available.

**Key learning points/Conclusion:**

Despite this, for the child/young person experiencing not only pain, but disgust, shame and body perception disturbance, the argument for amputation can be strong, and has to be considered.  We present the evidence to date and share the complexity of such discussions, focusing on the experience gained from these cases.

